# Evaluation of Inferior Alveolar Nerve Regeneration by Bifocal Distraction Osteogenesis with Retrograde Transportation of Horseradish Peroxidase in Dogs

**DOI:** 10.1371/journal.pone.0094365

**Published:** 2014-04-14

**Authors:** Yosuke Shogen, Emiko Tanaka Isomura, Mikihiko Kogo

**Affiliations:** 1 First Department of Oral and Maxillofacial Surgery, Osaka University, Graduate School of Dentistry, Suita, Japan; 2 Unit of Dentistry, Osaka University Hospital, Suita, Japan; University of North Carolina at Chapel Hill, United States of America

## Abstract

**Background:**

Bifocal distraction osteogenesis has been shown to be a reliable method for reconstructing segmental mandibular defects. However, there are few reports regarding the occurrence of inferior alveolar nerve regeneration during the process of distraction. Previously, we reported inferior alveolar nerve regeneration after distraction, and evaluated the regenerated nerve using histological and electrophysiological methods. In the present study, we investigated axons regenerated by bifocal distraction osteogenesis using retrograde transportation of horseradish peroxidase in the mandibles of dogs to determine their type and function.

**Methods and Findings:**

Using a bifocal distraction osteogenesis method, we produced a 10-mm mandibular defect, including a nerve defect, in 11 dogs and distracted using a transport disk at a rate of 1 mm/day. The regenerated inferior alveolar nerve was evaluated by retrograde transportation of HRP in all dogs at 3 and 6 months after the first operation. At 3 and 6 months, HRP-labeled neurons were observed in the trigeminal ganglion. The number of HRP-labeled neurons in each section increased, while the cell body diameter of HRP-labeled neurons was reduced over time.

**Conclusions:**

We found that the inferior alveolar nerve after bifocal distraction osteogenesis successfully recovered until peripheral tissue began to function. Although our research is still at the stage of animal experiments, it is considered that it will be possible to apply this method in the future to humans who have the mandibular defects.

## Introduction

Mandibular defects range from isolated segmental defects to large areas of extensive bone loss including the entire jaw. These are often congenital, though they can also result from trauma, infection, or resection of a benign or malignant tumor.

Bifocal distraction osteogenesis (BDO) is known to be a reliable method for reconstructing missing segments of bone, and several experimental and clinical studies have been presented [1]–[16]. Morphologically different from long bones of the extremities, mandibular bone tissues are involved with the inferior alveolar nerve (IAN), artery, and vein. BDO is often an excellent treatment option, because both hard and soft tissues can be reconstructed. Thus, we speculate that nerve regeneration with bifocal distraction osteogenesis will improve sensation in the alveolar ridge and gingiva to normal along with IAN regeneration. We previously reported histological and electrophysiological findings of regenerated IANs [17], [18]. In the present study, we regenerated the IAN in dogs by mandibular bifocal distraction osteogenesis and evaluated its function based on the retrograde transport of horseradish peroxidase (HRP).

## Methods

### Animals

We performed BDO procedures in 11 healthy adult male beagle dogs (age 9–14 months, weight 8–11 kg). The right side of the mandible in each animal was used as the operation side, while the other side served as the control (non-operated) side. The animals were housed in separate cages, and given solid food (Oriental Yeast Co., Tokyo, Japan) and water *ad libitum*. All experimental protocols were reviewed and approved by the Intramural Animal Care and Use Committee of Osaka University Graduate School of Dentistry prior to beginning the experiments.

### Surgical Procedure

The teeth of the left side of the mandible were extracted, while the canine and incisor teeth were preserved. Seven days later, surgery was performed under general anesthesia by an intramuscular injection of medetomidine (0.02 mg/kg) and midazolam (0.3 mg/kg), with an intraperitoneal injection of sodium pentobarbital (5 mg/kg) given 15 minutes later. Next, with the animal in a supine position, endotracheal intubation was started.

**Figure 1:**
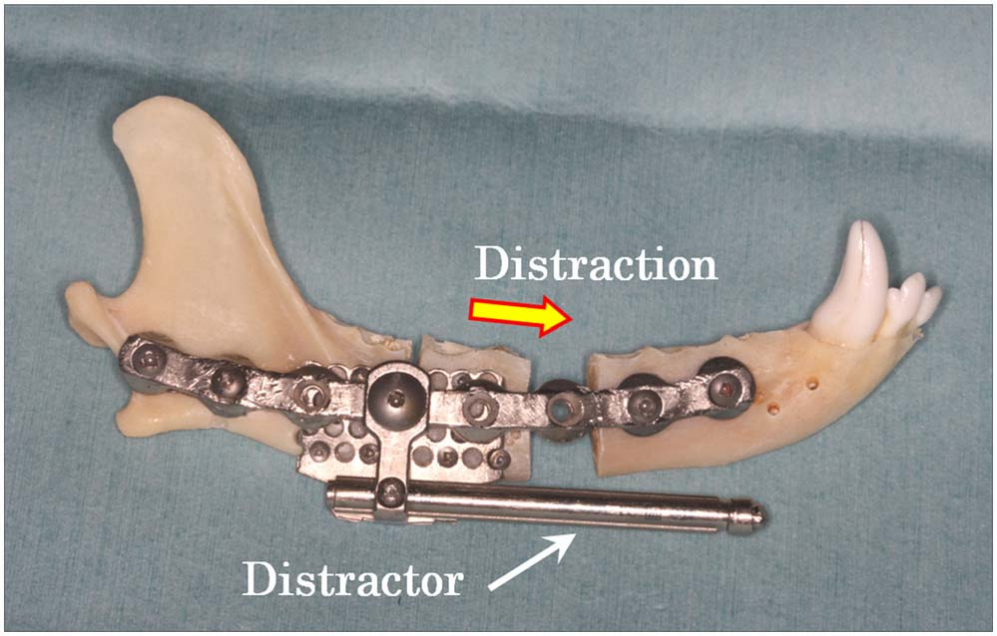
Clinical image showing surgical procedures. The IAN was not cut on the proximal side of the transport segment. Bifocal distraction was begun at a rate of 1/day and continued for about 10 days until the transport bone segment contacted the distal segment.

A skin incision was made along the inferior border of the mandible, extending from the angle to the level of the canine tooth in each dog. Once the periosteum was exposed, it was incised and carefully elevated only on the buccal side. The lingual side was not elevated, as it is necessary for blood supply to transport the bone segment. An 8-hole titanium reconstruction plate (ThreadLock System; KLS Martin, Jacksonville, America) was pre-molded and applied to the inferior aspect of the mandibular body. Two mesh distractor plates (ThreadLock Transport Distractor,; KLS Martin) were also applied to the mandibular body, inferior to the reconstruction plate, and an osteotomy line was formed. One of the mesh plates was placed in the proximal position, while the other was placed just next to the distal part of the bone of the previous plate that was to become the transport bone segment. The plates were then removed and a critical-sized mandibular defect including the IAN was created by excising 10 mm of bone with a reciprocating saw at a point 15 mm proximal to the mental foramen. An additional osteotomy was performed proximally to create a transport bone segment (the transport disk) of approximately 10 mm in size. Great care was taken to elevate the periosteum on the lingual aspect of the mandible and protect it while cutting bone, and also avoiding the IAN. The IAN was not cut at the proximal side, because the transport disk was to be transported along with the central connection of the nerve. The reconstruction plate and distractor were then reapplied, and the transport disk was fixed to one of the mesh plates of the distractor (Fig. 1). The activator of the distractor was exposed from the inferior border of the mandible to outside the oral cavity.

All dogs were allowed to heal for 7 days, after which distraction of the transport bone segment was begun at a rate of 1 mm/day and continued for about 10 days, until the transport bone segment made contact with the distal segment beyond the bone defect. Once distraction was completed, the lengthening device of the distractor was left in place for 28 days to allow for consolidation of the newly formed bone. Thereafter, the distractor and reconstruction plate were replaced with a new 8-hole titanium reconstruction plate (ThreadLock System; KLS Martin) under general anesthesia. At that time, the osseous tunnels of each end of the bone were realigned in cases with large misalignment.

### Labeling Primary Neurons in the Trigeminal Ganglion with HRP

To assess recovery of nerve function and the type of recovered axons, labeling of primary neurons in the trigeminal ganglion (TG) with HRP was performed under general anesthesia provided by an intramuscular injection of medetomidine and midazolam, with an intraperitoneal injection of sodium pentobarbital given before harvesting nerve samples at 3 (n = 6) and 6 (n = 5) months after the first operation. A skin incision was made along the inferior border of the mandible extending from the angle to the level of the canine tooth in each dog. On the control side, the periosteum was incised and elevated, and the mental nerve was exposed at the mental foramen. On the distracted side, the reconstruction plate was removed and the mental nerve was exposed at the mental foramen.

On both sides, the mental nerves were cut at about 5 mm distal from the mental foramens. The proximal stump of the nerve was inserted into a piece of fine polyethylene tubing (SURFLO, TERUMO, Tokyo, Japan), which was filled with 10 µl of 15% HRP (Sigma type VI; Sigma Aldrich, St. Louis, USA), and sealed to prevent leakage [19]. Ninety minutes later, the wounds were irrigated with saline and closed. Three days later, the dogs were euthanized with perfusion through the common carotid artery. Under general anesthesia provided by an intramuscular injection of medetomidine and midazolam, and an intraperitoneal injection of sodium pentobarbital, the bilateral carotid arteries and external jugular veins were exposed. The right carotid artery was catheterized with an 18-G intravenous cannula (SURFLO, TERUMO, Tokyo, Japan) and the left external jugular vein was sectioned at the proximal side of the branch site of the lingual vein. The perfusion was initiated by 0.1 M phosphate buffer (PB) at pH 7.4 until blood was cleared, followed by fixing with 4% paraformaldehyde and 0.5% glutaraldehyde in 0.1 M PB at pH 7.4. The perfusion was continued until the head and face were firm and cool to touch, and the tongue and oral mucosa had turned white. After the mandibular nerve was sectioned at a site 5 mm proximal from the mandibular foramen, the mandibular bone was extirpated en bloc to ensure bone recovery after BDO. Complete bone regeneration using BDO was successfully completed in all dogs. The ophthalmic and maxillary nerves were sectioned at a side 5 mm distal from the TG, then the mandibular nerve was sectioned at a site 20 mm distal from the TG so as to be distinguished from the other nerves. Finally, the TG was extirpated.

All TG sections were reacted with 3,3′,5,5′-tetramethylbenzidine (TMB, Sigma Aldrich, St.Louis, America) as the HRP reaction product. The extirpated TG was fixed for 12 hours in 1% paraformaldehyde and 1% glutaraldehyde in 0.1 M PB at pH 7.4. The ophthalmic, maxillary, and mandible nerves were sectioned at a site 3 mm distal from the TG. The proximal sectional site was 3 mm proximal from the TG and the epineurium was removed. The TG was placed in 20% sucrose and 4% paraformaldehyde in 0.1 M PB at pH 7.4 at 4°C for 2 days, followed by 30% sucrose in 0.1 M PB at pH 7.4 at 4°C for 24 hours, then frozen and cut serially and horizontally into sections with a thickness of 50 µm, and stored in 0.1 M PB at pH 7.4. The sections were washed in distilled water and processed in TMB solution (sodium nitroprusside dehydrate 200 mg, acetate buffer 10 ml, TMB 10 mg, absolute ethanol 5 ml, distilled water 185 ml) for 20 minutes at room temperature in a dark field. Addition of 20 µl of 30% hydrogen peroxide water to the medium stopped the reaction and the sections were washed in 5% acetate buffer (5-fold dilution of 1M sodium acetate 200 ml, 1N hydrogen chloride 190 ml, distilled water 610 ml) in a dark field.

When that process was finished, the sections were placed onto MAS-coated slides (MATSUNAMI GLASS IND., Osaka, Japan), dried overnight at 4°C, dehydrated in an ascending series of ethanol, cleared in xylene, and mounted with a water soluble mounting medium (MOUNT-QUICK; Daido Sangyo Co., Saitama, Japan). All sections were examined with a light microscope (DM-2000; Leica Microsystems, Wetzlar, Germany). Cells selected for measurements fulfilled 1 of 2 criteria; they were either homogeneously labeled with HRP or had vesicles inconsistently labeled with HRP. Cell diameter was calculated as the mean between the longest diameter and a diameter perpendicular to, then dividing the longest diameter into two equal halves.

## Results

**Figure 2:**
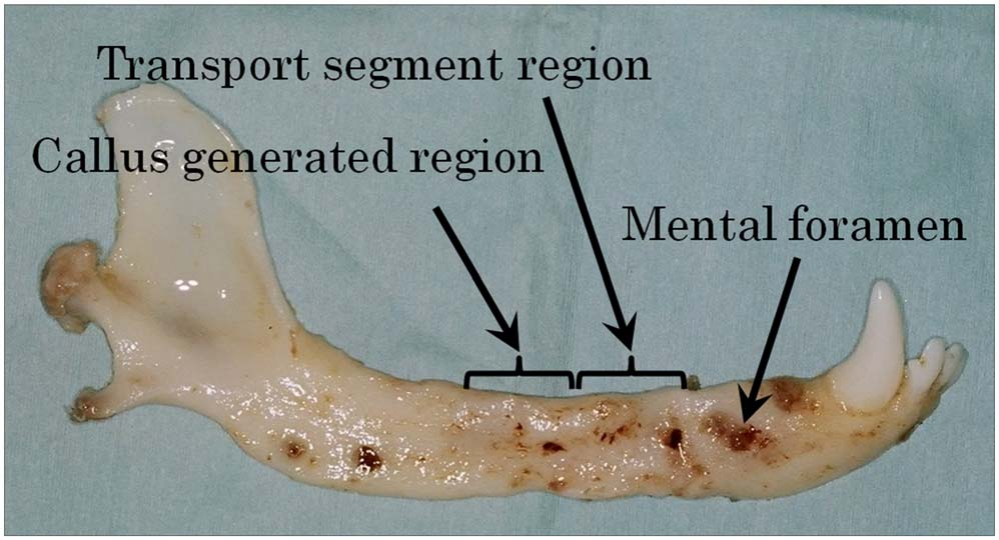
Extirpated mandibular bone. After removing the reconstruction plate and distractor, the transport bone segment was in contact with the distal segment and generated callus was observed on the proximal side of the transport segment.

**Figure 3:**
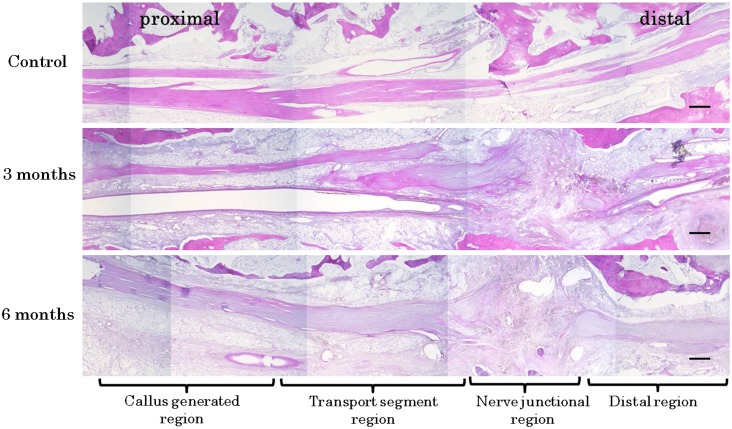
Histologic findings in total area at 3 months and 6 months (hematoxylin-eosin staining). Histologic findings showed continuous nerve tissue from the proximal to distal side in all groups. Control: (flip horizontal image). The IANs were observed longitudinally from proximal side to distal side. 3 months: Fibrous connective tissue containing a number of blood capillaries was apparent in the nerve connection area. 6 months: Compared with that of 3 months, blood capillaries in fibrous connective tissue of the nerve connection area was decreased. Scale bar = 1000 µm.

Complate bone regeneration using BDO was successfully completed in all dogs (Fig. 2). Histologic findings showed continuous nerve tissue from the proximal to distal side (Fig. 3). Fibrous connective tissue containing a number of blood capillaries was apparent in the nerve connection area at 3 months and 6 months. However, compared with that of 3 months, blood capillaries in fibrous connective tissue of the nerve connection area was decreased at 6 months.

**Figure 4:**
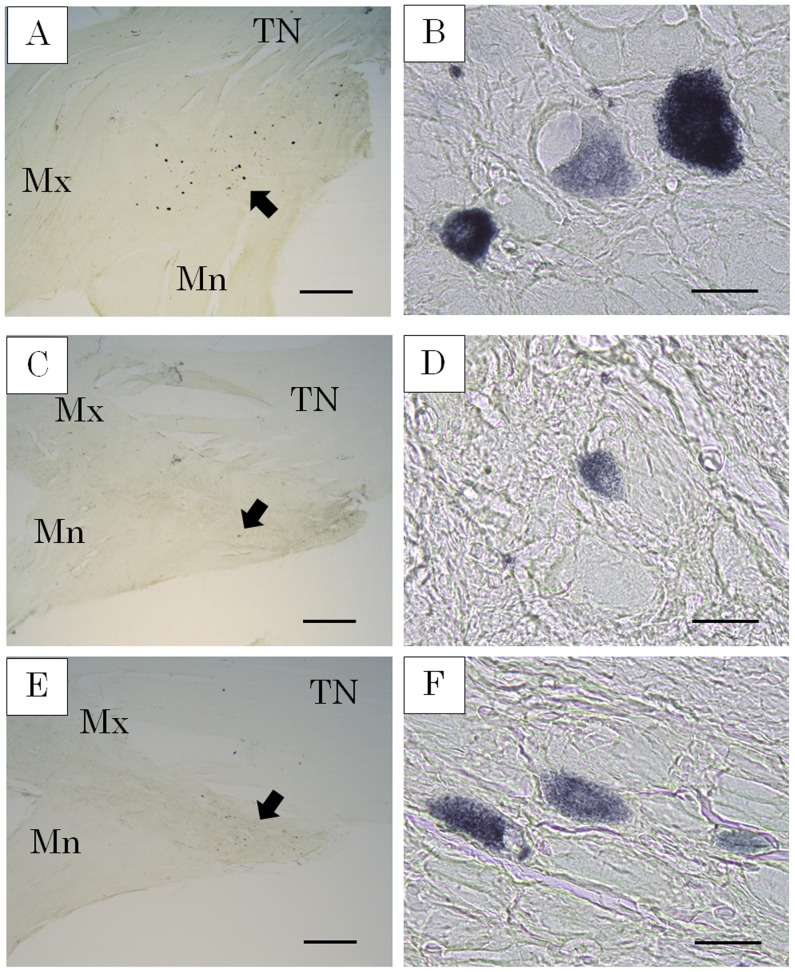
HRP-labeled neurons (TMB staining). HRP-labeled neurons were frequently seen from the base of the mandible nerve to the posterolateral area on both sides of the TG. A. TG on control side. B. HRP-labeled neurons on control side. C. TG after 3 months. D. HRP-labeled neurons after 3 months. E. TG after 6 months. F. HRP-labeled neurons after 6 months. TN: trigeminal nerve, Mx: maxillary nerve, Mn: mandibular nerve Scale bar = 1000 m in A, C, E, and 50 m in B, D, F.

**Figure 5:**
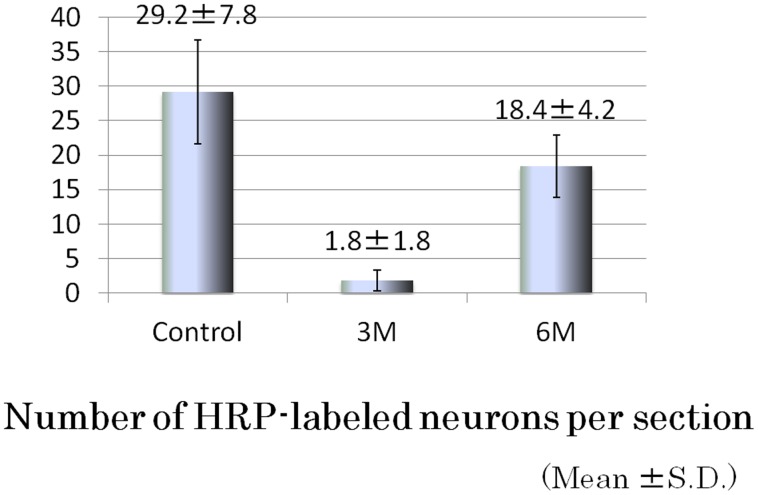
Average number of HRP-labeled neurons per section. The number of HRP-labeled neurons per section was low at 3 months and then increased to about 60% of the control side at 6 months.

**Figure 6:**
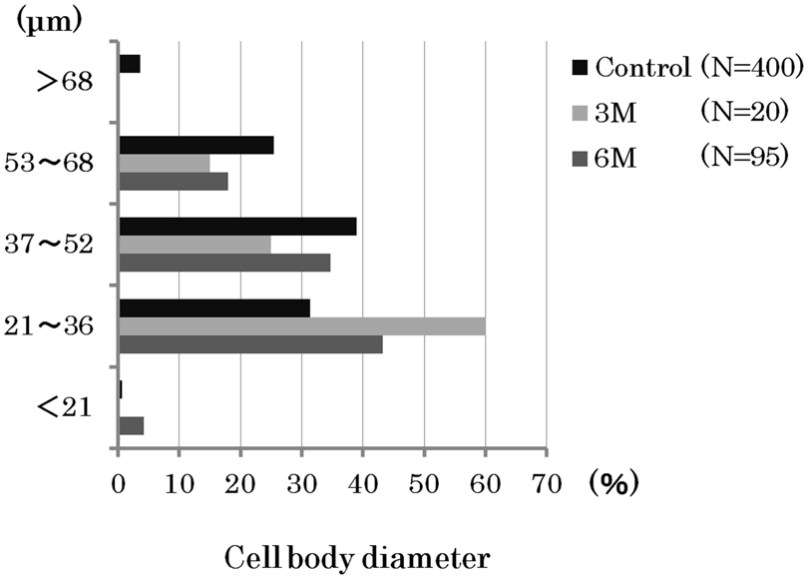
Frequency of distribution of HRP-labeled cell body diameters. The cell body diameter of HRP-labeled neurons on the control side ranged from 20∼70 µm (n = 400), with the majority sized from 37∼52 µm. After 3 months, they ranged from 26∼65 µm (n = 20), with the majority sized from 21∼36 µm, and after 6 months ranged from 19∼68 µm (n = 95), with the majority sized from 21∼36 µm. The size became reduced in the regenerated nerves.

HRP-labeled neurons were observed in the TG of all control sides (n = 6) at 3 months and the distraction side of 5 of 6 dogs at 6 months after the first operation. HRP-labeled neurons were frequently seen from the base of the mandible nerve to the posterolateral area on both sides of the TG (Fig. 4). There were few HRP-labeled neurons per section at 3 months, while they were increased by approximately 60% at 6 months on the control side (Fig. 5). The cell body diameter of the HRP-labeled neurons on the control side ranged in size from 20∼70 µm (n = 400), with the majority from 37∼52 µm. On the distraction side, the cell body diameter of HRP-labeled neurons after 3 months ranged from 26∼65 µm (n = 20), with the majority from 21∼36 µm. After 6 months, the cell body diameter of HRP-labeled neurons on the distraction side ranged from 19∼68 µm (n = 95) and the majority was sized from 21∼36 µm (Fig. 6).

## Discussion

A number of reports have been presented regarding the function of the IAN after monofocal distraction osteogenesis of the mandible [20]–[23]. Those revealed that IAN function after that procedure temporarily decreased, then gradually recovered and finally became nearly normal without surgical injury.

The IAN is involved with the mandibular bone, thus it is expected that BDO for a mandibular defect will lead to not only mandibular bone reconstruction, but also stretch the IAN and recover its continuous length. Several studies of mandibular bone reconstruction with BDO have been performed, though none has performed a functional evaluation of the IAN after BDO prior to the present experiment [1], [2], [4], [5], [10], [11], [14], [16].

In our previous study, histological and electrophysiological findings showed that it is possible to reconstruct a segmental mandibular defect and recover the IAN with BDO [17], [18]. When performing the electrophysiological examination, we found that it was possible to evoke action potentials for leakage even when the IAN had not recovered until the mental foramen. Although recovery of the IAN was confirmed by histological results, we found it necessary to understand how the axons of the regenerated IAN recovered in a continuous manner to the mental foramen.

In the present study, HRP-labeled neurons were observed in the TG of the distraction side and it was confirmed that the regenerated IAN was running from the TG to the mental foramen. HRP-labeled neurons were also frequently seen from the base of the mandible nerve to the posterolateral area on the TG, as noted in previous reports [24], [25]. At 3 months after the procedure, HRP-labeled neurons were few in number, while that population recovered to about 60% of the control side after 6 months. As for the diameter of the cell body of the HRP-labeled neurons, smaller cells occupied a greater portion in the control side as compared to the distraction side after 6 months. Following IAN injury, it has been reported that the number of TG cells was the same or decreased, while that latter study also found no differences regarding ganglia cell size and shape difference between nerve-sectioned and control ganglia [26], [27]. The major portion of small-diameter TG neurons are thought to function as sensory neurons with C-fibers [28]–[30]. Therefore, we speculate that regeneration nerves peripheral to the nerve junctional region increase over time and largely consist of nociceptor fibers.

We can not evaluate recovered somatic sense in dogs, though consider that all somatic sense decreased and nociception takes precedence over touch, pressure, and temperature in the regenerated nerve. When adapted for humans, the present method may lead to somatic sense recovery, though it could also cause dysesthesia or allodynia.

Presently, resectioned peripheral nerves can be clinically regenerated with nerve autografts and polyglycolic acid (PGA)-collagen tubes. As for regeneration of peroneal nerves of dogs, regenerated axons with nerve autografts were about 75% of normal nerves and regenerated axons with PGA tubes were about 80% [31]. In the present study, using HRP labeling we calculated that the regenerated axons comprised about 60% of the population at 6 months after surgery. Another study determined the number of regenerated nerves in the nerve implanted area using a transmission electron microscope [31]. These results should not be simply compared as different means of evaluation, as it seems that the success rate of our study was lower. Improvements in the present method are required to improve the success rate.

Although clinical application of mandibular bone recovery with BDO needs refinement, our method may provide both morphological and functional recovery of the mandible, leading to quality of life improvements for patients with mandibular defects.

## Conclusion

The present results indicate that the inferior alveolar nerve after bifocal distraction osteogenesis can successfully recover until peripheral tissue resumes function. Although our research is still at the stage of animal experiments, it is considered that it will be possible to apply this method in the future to humans who have the mandibular defects.
